# Adherence to weekly anal self-examination among men who have sex with men for detection of anal syphilis

**DOI:** 10.3389/fmed.2022.941041

**Published:** 2022-08-01

**Authors:** Ei T. Aung, Christopher K. Fairley, Jason J. Ong, Tiffany P. Phillips, Julien Tran, Marcus Y. Chen, Kate Maddaford, Eric P. F. Chow

**Affiliations:** ^1^Melbourne Sexual Health Centre, Alfred Health, Melbourne, VIC, Australia; ^2^Central Clinical School, Faculty of Medicine, Nursing and Health Sciences, Monash University, Melbourne, VIC, Australia; ^3^Centre for Epidemiology and Biostatistics, Melbourne School of Population and Global Health, The University of Melbourne, Melbourne, VIC, Australia

**Keywords:** anal self-examination, syphilis, men who have sex with men (MSM), adherence, anal syphilis, weekly exam, feasibility

## Abstract

**Background:**

Men who have sex with men (MSM) practicing exclusively receptive anal sex are more likely to present with secondary than primary syphilis, implying primary anorectal lesions may be missed. If men could detect anorectal lesions by regular anal self-examination, the duration of infectiousness could be reduced. This study aimed to examine adherence to weekly anal self-examination.

**Method:**

We conducted a longitudinal feasibility study examining the adherence to weekly anal self-examinations among MSM attending a sexual health clinic in Melbourne, Australia between December 2020 and June 2021. Adherence to weekly anal self-examinations over 12 weeks was assessed from a logbook and 4-weekly surveys. Participants who identified abnormalities in their anus were recommended to seek medical review.

**Results:**

Of the 30 men who completed the study, anal self-examination was performed at least weekly for 308 of 360 person-weeks (86% of the weeks, 95% CI: 82–89). The mean adherence was 3.6 (95% CI: 3.3–3.9) examinations per 4-weeks per person in Weeks 1–4, 3.5 (95% CI: 3.1–3.8) in Weeks 5–8 and 3.3 (95% CI: 2.9–3.7) in Weeks 9–12 (*P*_*trend*_ = 0.06). Six men (20%, 6/30) were seen for medical review after they identified abnormalities, whilst eight men (27%, 8/30) reported abnormalities, but did not seek medical review. No participants were diagnosed with syphilis during the study period.

**Conclusion:**

We conclude that men adhered well to weekly anal self-examination. Therefore, it is feasible to trial this as a routine practice among MSM. Future studies should investigate possible reductions in adherence over time and ways to increase medical review for abnormalities that men find.

## Introduction

High and rising rates of syphilis among gay, bisexual, and other men who have sex with men (MSM) are occurring despite substantial public health interventions and strategies to improve syphilis control (1–4). These public health interventions include widespread testing, contact tracing, contact treatment and behavioral interventions such as promoting condoms. The limited success in syphilis control with the existing public health interventions and strategies highlights the need for additional interventions.

Regular syphilis screening is one option for potentially improving syphilis control. The current guidelines for screening of human immunodeficiency virus (HIV) and sexually transmitted infections (STIs) in Australia recommend 3 monthly screening (including syphilis serology) for sexually-active MSM and also for MSM taking HIV pre-exposure prophylaxis (PrEP) (5). An Australian study found a substantial proportion of infectious syphilis cases (58% of primary syphilis and 44% of secondary syphilis) were diagnosed between the 3-monthly routine clinic visits among MSM taking PrEP (6). The findings indicate that even 3-monthly screening is insufficient to detect all cases and a substantial number of cases occur between these visits. Therefore, additional measures for the early detection of syphilis are warranted.

One strategy to shorten the duration of infectious of syphilis cases has been to improve the patients' recognition of syphilis symptoms and encourage early presentation to health care. However, recent research has indicated some primary syphilis lesions may be in anatomical positions that make their detection difficult. A study examining the sexual position and staging of syphilis reported that MSM who practiced exclusively receptive penile-anal sex were four times more likely to present with secondary syphilis than those who practiced exclusively insertive penile-anal sex, suggesting primary anorectal lesions were often missed leading to secondary syphilis (7). If there was a way of detecting the missed ano-rectal primary syphilis lesions, then progression to secondary syphilis would be prevented. Preventing secondary syphilis is important not only because there is substantial shedding of *T. pallidum* in this stage (8) but also because secondary syphilis is associated with systemic illnesses and complications such as ocular syphilis and neurosyphilis. We hypothesized that if men examine their anus regularly (e.g., once a week), men might be able to detect primary anorectal lesions and therefore seek medical care and receive timely diagnosis and treatment before progressing to secondary syphilis, thereby reducing infectiousness and further transmission.

Regular anal self-examination is a new concept for detecting primary syphilis but has been investigated among MSM living with HIV to detect anal cancer at an early stage (9). These studies have shown that anal self-examination is acceptable for the detection of anal cancer (9, 10). Furthermore, qualitative, and quantitative studies have shown that MSM are willing to perform anal self-examinations for detecting anal syphilis (11, 12). Before we examine the effectiveness of anal self-examination for early anal syphilis detection, we first need to examine whether weekly anal self-examination is feasible as a regular practice among MSM.

We designed this feasibility study to explore adherence to weekly anal self-examination. The primary aim of this study was to investigate the adherence to weekly anal self-examination, and the secondary aim was to examine the proportion of men returning for medical review when they detect abnormality in the anus during anal self-examination.

## Materials and methods

### Anal self-examination

Anal self-examination in our study was defined as inserting a finger into one's anus, feeling around the anal canal (360°), and using a mirror to check the anus and surrounding area for any abnormalities.

### Study population and recruitment

This was a cohort study conducted at the Melbourne Sexual Health Center (MSHC) between 1st December 2020 and 17th June 2021. The last participant was recruited on 10th March 2021 and the data collection of the last participant was on 17th June 2021. MSHC is a public sexual health clinic in Victoria, Australia, which provided approximately 50,000 consultations in 2019. To be eligible in this study, men must be aged 18 years or above, identified as cis-male who had had receptive penile-anal sex with another man in the past 12 months and planned to reside in Victoria, Australia in the next 3 months. Men who only had insertive penile-anal sex or men who had been performing weekly anal self-examination were not eligible. We aimed to recruit 30 MSM including ten men living with HIV, ten men taking PrEP, and ten men not living with HIV and not taking PrEP.

Eligible men were identified by clinicians and were referred to the research team. One of the research team members (EA, KM, ER) met with the potential participant on the same day or arranged another appointment if they were unavailable on the day. A member of the research team obtained written informed consent from participants before the start of the study. We sent the study website link *via* SMS to the participants on the day of enrolment. We explained the process of anal self-examination using anal self-examination instruction (Supplementary Figure 1) and provided the instruction sheet to the participants. The study website contained information about anal self-examination, an animated video about the instruction on how to perform anal self-examination, contact details of the research team if the participant found any abnormalities during anal self-examination, a downloadable logbook and anal self-examination instructions.

### Study protocol

#### Baseline

At recruitment, demographics (e.g., age, gender), sexual practices (e.g., sexual orientation and position of anal sex), and medical history (e.g., HIV status, PrEP use, past history of syphilis and previous experience of anal self-examination) were collected using a self-administered questionnaire. Participants were asked to perform weekly anal self-examination over 12 weeks and record their activity on a logbook (electronic or paper-based) (Supplementary Table 1).

#### Follow-up

Participants were asked to complete another three surveys at Weeks 4, 8, and 12. The follow-up surveys collected data on adherence to anal self-examination, and abnormal findings and problems encountered during anal self-examination. The Week 12 survey also collected the willingness to perform anal self-examination in the future. In order to differentiate anal self-examination from At the end of Week 12, the participants were also asked to return the logbook and complete the last survey. Participants were given an AU$50 (~US$22) gift card for remuneration at the end of the study.

#### Adverse event or abnormal findings

Participants were advised to contact the research team *via* email or phone if they identified any abnormal findings in their anus that concerned them. Once the participant contacted the research team, an appointment was arranged at a time suitable for the participant to see a study doctor, which was usually about within 1–3 days. The study doctor examined the participant's anus to review the abnormal findings and the participant also had a serological test for syphilis on the day. A polymerase chain reaction (PCR) test for *Treponema pallidum* was also performed if any anal lesions were present. If the participants could not attend MSHC for review, the participants then opted to have a review with a general practitioner, and they would inform the research team of the review and diagnosis.

#### Syphilis diagnoses

Syphilis test results from the participants over the 12-week study period were also extracted from the electronic medical records at the Melbourne Sexual Health Center. In addition, we collected syphilis test results data 12 weeks after they completed the final Week 12 visit.

### Outcomes

The primary outcome was self-reported adherence to anal self-examination (i.e., whether men performed weekly anal self-examination) over a 12-week period. The secondary outcomes were (1) adverse events where the participants identified abnormal findings during anal self-examination or (2) diagnosed with syphilis during the study period. The adverse events were defined as any abnormal findings from anal self-examination. There were two categories of adverse events: (1) participants were concerned about abnormal findings and requested a medical review, and (2) participants were not concerned about abnormal findings and did not request a medical review. In the group of men who requested a medical review due to abnormal findings, the events were reported to the research team *via* email or phone contact, or the events were noted from the medical records at MSHC when they returned for review. If the participants sought medical review with their general practitioners instead, the participants would inform the research team of the outcomes. In the group of men who had abnormal findings but did not request a medical review, the events were noted from the surveys and followed up by email or phone contact with the participants.

### Ethics approval

Ethical approval was obtained from the Alfred Hospital Ethics committee, Melbourne, Australia (Project 603/20).

### Sample size

We designed this study to provide reasonably precise 95% confidence intervals (CI) around the primary aim of adherence to weekly examinations over 12 weeks. With 360 weeks of observation, we estimated the 95% CI will be ± 5% of the mean proportion (13).

### Statistical analysis

The primary outcome of the adherence to weekly anal self-examination was the proportion of the number of weeks expressed in person-time. It was defined as the number of weeks where the participants had performed anal self-examination divided by the total number of weeks in the study period for total study participants. The outcome was expressed in person-weeks. The 95% CI of the proportion were calculated using the binomial exact method. We calculated the mean with 95% CI of the frequency of anal self-examination every 4 weeks per person and we examined the temporal trend on anal self-examination per 4-week using linear regression analysis.

The secondary outcomes were summarized using descriptive statistics by reporting the proportion of men who reported abnormal findings out of the total number of participants. The adverse events were calculated for the proportion of men who reported abnormal findings and requested medical review (either at MSHC or with their general practitioners), and for the proportion of men who reported abnormal findings and did not request medical review.

Kaplan-Meier survival curves were constructed to present the cumulative proportion of men who first developed adverse outcomes and received medical review. All statistical analyses were conducted using STATA 16 (StataCorp LLC, Texas, USA).

## Results

There were 36 men recruited from December 2020 to March 2021, and the last participant finished the study in June 2021. Of the 36 men, four never completed the baseline surveys and were classified as lost to follow-up. One completed only the baseline survey, and one withdrew from the study at Week 9. A total of 30 men who completed baseline and follow-up surveys were included in the final analysis (Supplementary Figure 2). All 30 men tested negative for active syphilis at baseline.

Table 1 presents the demographic characteristics and sexual practices of 30 participants. The age of the participants ranged from 19 to 55 years (median 32 years, IQR: 27-41). Among the 25 men (83%) who had previously inserted their fingers in their anus, the median frequency of performing this was once per 4 weeks (IQR: 0.3–4).

**Table 1:** Demographic characteristics and sexual practices among 30 participants at baseline.

**Demographic characteristics and sexual practices**	**Number of participants, Percentage (%)**	
Age (median, interquartile range) (years)	32 (IQR: 27–41)	
**Gender**		
Men	30	100%
**Sexual orientation**		
Men who have sex with men	30	100%
Men who have sex with men and women	0	0%
**HIV and PrEP**		
Living with HIV	8	27.0%
Taking PrEP	11	37.0%
Not taking PrEP & not living with HIV	11	37.0%
**Anal sex position in the past 12 months**		
Receptive penile-anal sex only	14	47.0%
Receptive and insertive penile-anal sex	16	53.0%
**Past syphilis infection**		
Yes	9	30.0%
One infection	7	23.0%
More than one infection	2	7.0%
No	21	70.0%
**Condom use in the past 3 months** ^**∧**^	*N* =29	
Always	3	10.0%
Never	9	31.0%
Sometimes	17	59.0%
No anal sex	0	0.0%
**Ever inserted their fingers in their anus previously**		
Yes	25	83.0%
No	5	16.0%
Previous abnormalities reported by men who had inserted their fingers in their anus *	*N* = 25	
Yes^†^	9	36.0%
No	16	64.0%
Reasons for inserting their fingers in their anus among those who had performed previously*§	*N* = 25	
To check for symptoms of STI	15	58.0%
On recommendation by health professionals or friends/family/partners	4	15.0%
To check for abnormalities	3	12.0%
Pleasure/masturbation	2	8.0%
Hygiene	2	8.0%
Anal cancer screening	1	4.0%
Median sexual partners for receptive anal sex in the past 3 months*	4 [IQR: 1–7]	
Median frequency of anal self-examination* (per 4 weeks)	1 [IQR: 0.3–4]	
Mean frequency of anal self-examination* (per 4 weeks)	1 [SD ± 1.1]	

* Refers to the men who had previously performed anal self-examination prior to the enrolment in the study and the description in brackets were as described.

∧ The total number does not equal to 30 due to missing data.

§ Participants could provide more than one reason.

† Reported abnormalities included hemorrhoids, anal fissure, lump, dry skin, anal STI symptoms- bleeding, ulcer, discharge, pain.

IQR, interquartile range.

SD, standard deviation.

### Adherence to performing weekly anal self-examination

All 30 men were followed for 12 weeks and therefore provided 360 person-weeks of follow up. Anal self-examination was performed in 308 of 360 person-weeks (86% of the follow-up weeks, 95% CI: 82–89). Of the 30 men, 47% (*n* = 14) performed weekly anal self-examination over 12 weeks and 53% (*n* = 16) did not perform weekly anal self-examination.

The mean frequency of anal self-examination per 4 weeks changed from 3.6 (95% CI: 3.3–3.9) times in Weeks 1–4, to 3.5 (95% CI: 3.1–3.8) times in Weeks 5–8, and 3.3 (95% CI: 2.9–3.7) times in Weeks 9–12; however, this change was not statistically significant (*P*_*trend*_ = 0.06) (Supplementary Table 2).

### Reasons for not performing anal self-examination every week and motivators

Among the 16 men who did not perform anal self-examination every week, the commonly reported reasons were busy with work, study, or life, and forgetting to perform their anal self-examinations (Table 2). They reported having STI-related symptoms, increased sexual activity, and receiving a reminder as the top three motivators to perform anal self-examination regularly (Table 2).

**Table 2:** Sexual practices and anal self-examination including motivators and views of ways to improve adherence to anal self-examination.

	**Number**	**Percentage (%)**
Number of sexual partners for receptive anal sex in the past one month, median (IQR)	2 (1–4)	
Condom use in the past 3 months*^‡^	*N* = 28	
Always	4	14.0%
Sometimes	9	32.0%
Never	13	46.0%
No anal sex	2	7.0%
**Anal self-examination positions used by participants^**†**^^‡^**	*N* = 28	
Standing	6	21.0%
Squatting	7	25.0%
Standing & squatting	4	14.0%
Lying on the back	3	11.0%
Lying on the side	1	4.0%
Lying on the back & squatting	2	7.0%
Lying on the back & lying on the side	1	4.0%
Using more than 2 positions	4	14.0%
**Items used in anal self-examination^**†**^^‡^**	*N* = 59	
Lubricants	17	29.0%
Soap	14	24.0%
Water	18	31.0%
Gloves	2	3.0%
Mirrors	6	10.0%
None of the above	2	3.0%
**Location to perform anal self-examination^**†**^^‡^**	*N* = 38	
Shower	21	55.0%
Bed	5	13.0%
Bathroom/toilet	12	32.0%
**Reasons for non-adherence^**†**^^‡^**	*N* = 16	
Busy with work or study or life	11	69.0%
Forgot to perform anal self-examination	10	63.0%
Had not had anal sex	5	31.0%
No symptoms of STI	6	38.0%
Uncomfortable or difficult to perform	3	19.0%
Participants' views on the use of reminder system*^‡^	*N* = 30	
Weekly reminder system *via* SMS or a smartphone app or using phone as a reminder	11	34.0%
3-monthly SMS reminder	7	22.0%
Smartphone app (frequency not specified)	6	19.0%
Reminder not required	6	19.0%
Logbook	1	3.0%
Monthly reminder (method not specified by participant)	1	3.0%
Motivators to perform anal self-examination regularly*^‡^	*N* = 30	
Having symptoms of STI	6	22.0%
Increased sexual activity ^∧^	6	22.0%
Reminder	4	15.0%
Improvement in knowledge such as knowing what to look for and differentiating normal and abnormal	4	15.0%
Medical advice and recommendations or proven effective to be used as a screening for anal syphilis	3	11.0%
Does not need any motivation to perform regularly	4	15.0%

* Some missing data.

† Multiple options and total may exceed 100%.

∧ Increased sexual activity refers to increased sexual partners, engaging in high-risk anal sex such as condomless anal sex.

‡ Data were collected at Week 12.

### Preferences and intention to perform anal self-examination

At the end of the study, men reported standing, squatting or a combination of the two positions as the most common positions (81%, *n* = 17). The shower (55%, *n* = 21) was the most common location to perform anal self-examination. Most men (60%, *n* = 35) used water or lubricants to aid with anal self-examination (Table 2 and Supplementary Figure 3).

More than half (53%, *n* = 16) of participants felt that weekly anal self-examination was too frequent, while 37% (*n* = 11) reported that weekly anal self-examination was acceptable.

Most men (63%, *n* = 17) expressed their intention to continue performing anal self-examination if it were recommended in the future, while 23% (*n* = 7) were unsure if they would continue. A small number of men (7%, *n* = 2) chose not to continue to perform anal self-examination. Among those men who decided to continue their examinations, the mean preferred frequency was two times (95% CI: 1.4–2.6) per month.

### Abnormal findings during anal self-examination

**Figure 1:**
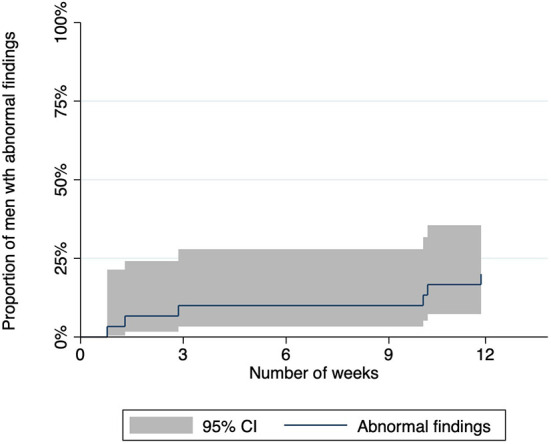
Graph showing the proportion of men who reported abnormal findings and requested medical review during the 12-week study period.

Of the fourteen men (47%, 14/30, 95% CI: 28–66%) who identified some abnormalities in their anus, six men (43%, 6/14, 95% CI: 18–71%) sought medical review (Figure 1); four at MSHC and two at their general practitioners. One of them presented twice with the same abnormalities and was diagnosed with recurrent herpes simplex (Table 3). Half of the men (50%, 3/6) who requested medical review reported abnormalities in Weeks 1–4, while the other half (50%, 3/6) reported abnormalities in Weeks 9–12.

**Table 3:** Reported abnormalities and clinical diagnoses among six men who presented for medical review after identifying an abnormality.

**Participant**	**Number of weeks first reported abnormality**	**Description of abnormality**	**Location of medical review**	**Diagnosis**	**Syphilis serology**	**Syphilis PCR**
3	12	Pain, bleeding	MSHC	Anal tear	Negative	Negative
12	11	Pain, bleeding, itch	GP	Rectal chlamydia	Negative	Not done
17	1	Lump	MSHC	Possible anal wart	Negative	Negative
23	2	Lump	GP	No abnormality found*	Negative	Not done
25	11	Itch, rash	MSHC	No abnormality found*	Negative	Negative
30	3, 12	Discomfort/pain	MSHC	Recurrent HSV-2	Negative	Negative

MSHC, Melbourne Sexual Health Center.

GP, General Practitioner.

HSV-2, Herpes Simplex Virus Type II.

* No abnormality found: Clinicians could not find any abnormalities at review; therefore, no diagnosis was given at the reviews.

Eight men (27%, 8/30 men, 95% CI: 12-46%) reported abnormalities in surveys but did not seek medical review (Supplementary Table 3). The symptoms reported were pain, lump, itch, dry skin, and blisters with anal pain being the most commonly reported symptoms (Supplementary Table 3).

### Syphilis diagnosis

None of the 30 men in the study were diagnosed with active syphilis at baseline (recruitment), at the completion of the study and also 3 months after completing the study (from reviewing the syphilis test results at MSHC).

## Discussion

This is the first study to examine adherence to weekly anal self-examination as a potential means of detecting anal syphilis in MSM. We found a high level of adherence (86% of the follow-up weeks) among the participants indicating that men might perform anal self-examination regularly if it were recommended. Almost half of the participants reported abnormalities in their anus but only about 40% of them sought medical review. Future studies with a longer follow-up period will be needed to assess the long-term adherence to anal self-examination and its sensitivity to detect anal syphilis. Nonetheless, finding abnormalities through anal self-examination demonstrate that it was feasible for men to perform anal self-examination and detect abnormalities.

There have been very limited studies examining the use of anal self-examination for early detection of anal syphilis and none examining adherence to these examinations (11, 12, 14, 15). Previous studies have involved both qualitative and quantitative surveys of MSM and found anal self-examinations to be highly acceptable (11, 12, 15). A survey of 574 MSM reported up to 68% of men had never performed anal self-examinations but were willing to perform them in the future (12). Consistent with these findings, we found that only a small proportion of MSM (7%) indicated that they did not want to continue performing anal self-examinations. Most of the 20 MSM in the qualitative interview study expressed their willingness to perform anal self-examination in the future if it were recommended by a health professional (11). Taken together with these findings, there is substantial evidence to support anal self-examination having a potential role in the detection of syphilis MSM.

Our study identified some issues that should be considered when designing future studies. Our findings suggest adherence to anal self-examinations may decline with time although this was not statistically significant. However future studies should consider incorporating SMS reminders in future studies on anal self-examination as most men in our study chose SMS messaging as a preferred reminder system. This finding was consistent with other studies showing SMS reminders increased the odds of adherence to intervention (16, 17). We also found that half of the abnormalities were reported at the start of the study suggesting men were identifying pre-existing abnormalities rather than new abnormalities that developed during the study period. This could potentially lead to an overestimation of the true incident abnormalities.

In our study, we found a high proportion of participants reporting abnormalities during self-examination yet none of the men had syphilis. Importantly, only about half of them sought medical review and we did not know the reasons why other participants did not seek medical review. We also did not know if anal syphilis was to occur, whether it would have an abnormality that could be detected by men during anal self-examination. Understanding these issues is going to be critical in determining whether syphilis can be detected earlier and whether it would be cost-effective or not.

### Limitations

There are several limitations to our study. First, clients attending sexual health clinics tend to be more health-conscious about their sexual health and therefore, there might be a selection bias with participants more likely to be adherent than the general MSM population. So, we may have overestimated adherence in our study, although high levels of acceptance were found in questionnaire studies (12). Second, the sample size was not sufficient for the estimates of our secondary outcomes such as the proportion reporting abnormalities. Third, self-reported bias in the adherence to anal self-examination might have occurred. There was a possibility of over-reporting and overestimating the adherence. Fourth, the study was conducted during the COVID-19 pandemic and lockdown periods resulting in some men reducing their sexual practices and changing the frequency of performing anal self-examination. In the surveys, men reported increased sexual activity and condomless sex would motivate them to perform anal self-examination more frequently. Therefore, COVID-19 pandemic restrictions could have affected their adherence (18–20).

### Conclusions

Overall, we conclude that men adhered well to weekly anal self-examination and therefore, they are feasible to trial as a routine practice among MSM.

## Data availability statement

The original contributions presented in the study are included in the article/Supplementary material, further inquiries can be directed to the corresponding author/s.

## Ethics statement

The studies involving human participants were reviewed and approved by the Alfred Hospital Ethics Committee, Melbourne, Australia (Project 603/20). The patients/participants provided their written informed consent to participate in this study.

## Author contributions

CF, EC, and EA designed and coordinated the study. JO, TP, and MC contributed to the development of the study protocol. EA, KM, and ER recruited the participants. EA conducted follow-ups, distributed the survey, coordinated the medical reviews, and drafted the 1st manuscript. EC and EA performed the analysis of the data and revised the manuscript. All authors reviewed and edited the manuscript and read and approved the final manuscript.

## Funding

This study was supported by an Australian National Health and Medical Research Council (NHMRC) Investigator Grant (GNT1172873) and there was no direct involvement by NHMRC in our study.

## Conflict of interest

EC was supported by an Australian NHMRC Emerging Leadership Investigator Grant (GNT1172873). CF was supported by an Australian NHMRC Leadership Investigator Grant (GNT1172900). JO was supported by an Australian NHMRC Emerging Leadership Investigator Grant (GNT1193955). EA was supported by Australian Government Research Training Program (RTP) scholarship from Monash University and Research Entry Scholarship from the Chapter of Sexual Health Medicine, Royal Australasian College of Physician. JT was supported by Australian Government Research Training Program (RTP) Scholarship from Monash University. The remaining authors declare that the research was conducted in the absence of any commercial or financial relationships that could be construed as a potential conflict of interest.

## Publisher's note

All claims expressed in this article are solely those of the authors and do not necessarily represent those of their affiliated organizations, or those of the publisher, the editors and the reviewers. Any product that may be evaluated in this article, or claim that may be made by its manufacturer, is not guaranteed or endorsed by the publisher.
