# Supplementary material for: Adherence to weekly anal self-examination among men who have sex with men for detection of anal syphilis
Source: Front Med (Lausanne). 2022 Aug 1;9:941041. doi: 10.3389/fmed.2022.941041 (PMC9376231; doi:10.3389/fmed.2022.941041)
Supplement: Supplementary file 1 [file Data_Sheet_1.pdf]

frontiers

Supplementary Material

Supplementary table 1. Schedule of study follow-up

	Start of the study	Week 4	Week 8	Week 12
Survey	x	x	x	x
Logbook<br><br>(To return after 12th week)	→	→	→	→

Supplementary Figure 1. Anal self-examination instruction (see pdf attached)

Supplementary Figure 2. Study flowchart

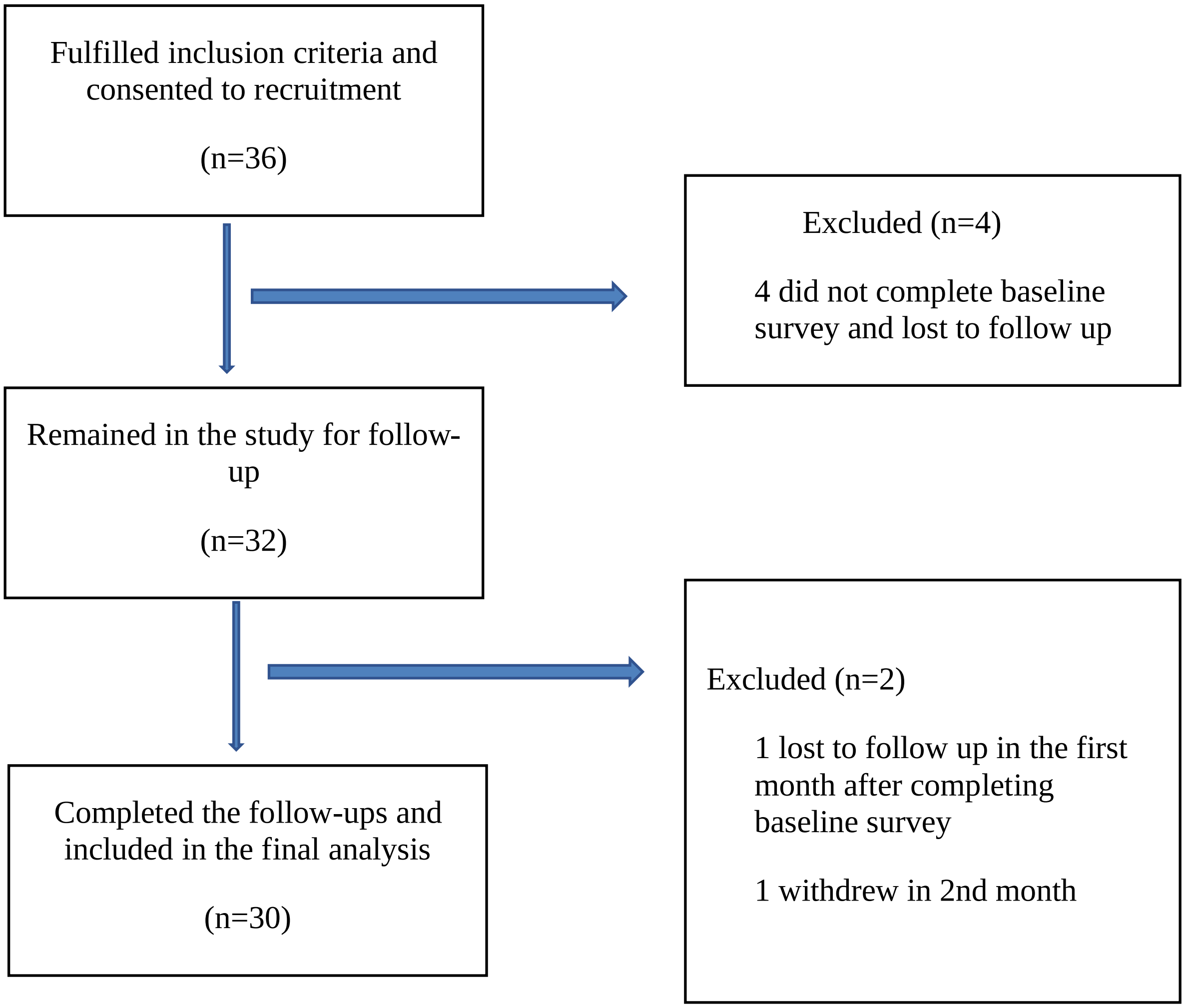

Supplementary Figure 3. Graph illustrating positions, items used as aids and locations to perform anal self-examination by men

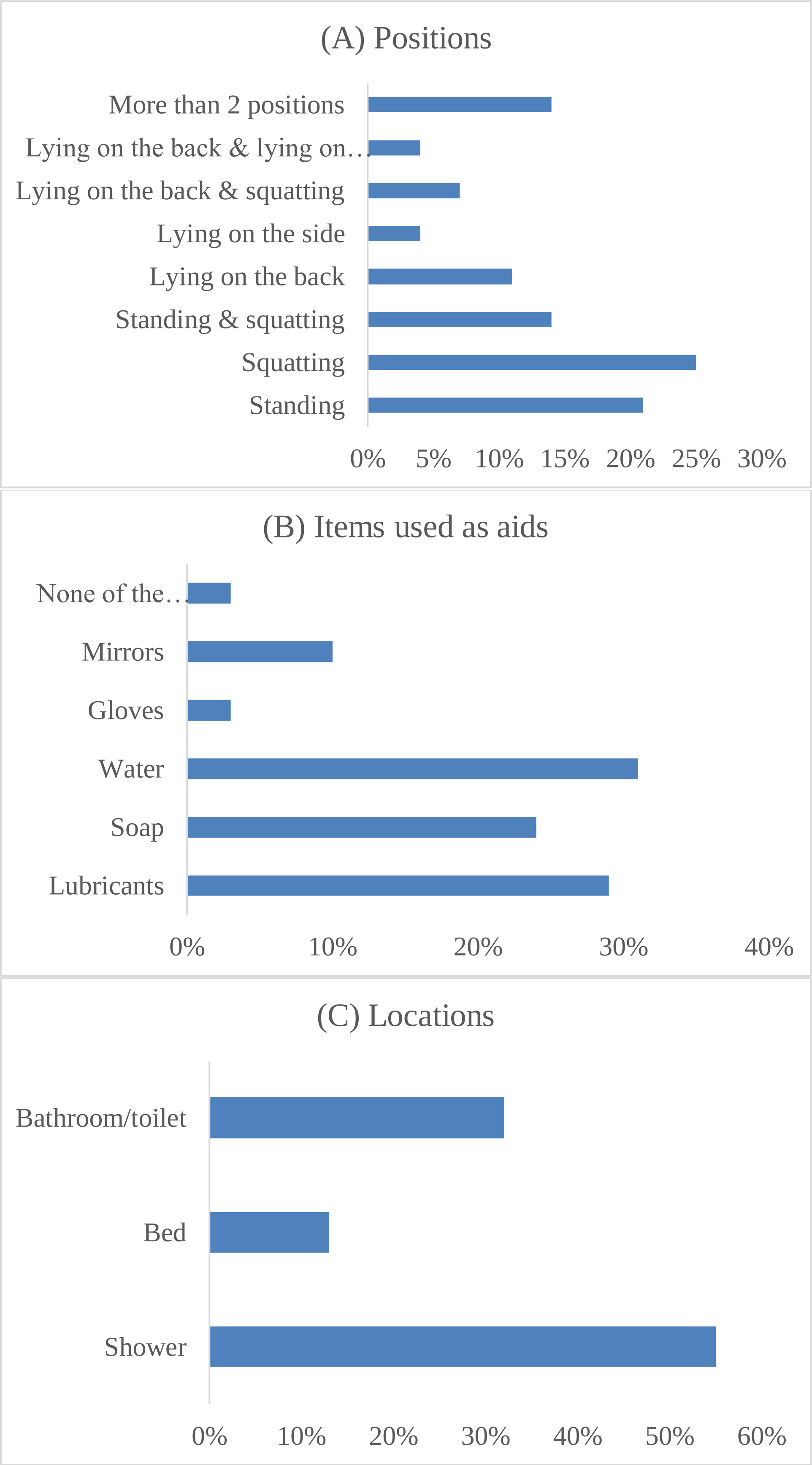
(A) Positions
More than 2 positions
Lying on the back & lying on…
Lying on the back & squatting
Lying on the side
Lying on the back
Standing & squatting
Squatting
Standing
0%
5%
10%
15%
20%
25%
30%
(B) Items used as aids
None of the…
Mirrors
Gloves
Water
Soap
Lubricants
0%
10%
20%
30%
40%
(C) Locations
Bathroom/toilet
Bed
Shower
0%
10%
20%
30%
40%
50%
60%

Supplementary Table 2. Mean anal self-examination frequency performed during 12 weeks study period

	Mean	Standard Deviation	[95% Conf.	Interval]
Week 1-4	3.6	0.8	3.3	3.9
Week 5-8	3.5	0.9	3.1	3.8
Week 9-12	3.3	1.1	2.9	3.7

ASE: anal self-examination

SD: standard deviation

Supplementary Table 3. Abnormalities reported by men who did not return for a medical review (N=8)

Abnormalities	Week 1-4	Week 5-8	Week 9-12	Total
Pain	2	1	4	4
Lump	1	2	1	2
Itch	1	1	1	2
Bleeding	0	0	0	0
Rash	0	0	0	0
Ulcers	0	0	0	0
Hemorrhoids (did not report abnormalities)	1	0	0	1
Others: dry skin, blisters	0	2	0	2

NB. Multiple abnormalities might be reported per participant.
