# Supplementary material for: Adherence to weekly anal self-examination among men who have sex with men for detection of anal syphilis
Source: Front Med (Lausanne). 2022 Aug 1;9:941041. doi: 10.3389/fmed.2022.941041 (PMC9376231; doi:10.3389/fmed.2022.941041)
Supplement: Supplementary file 2 [file Data_Sheet_2.pdf]

# Anal Self Examination

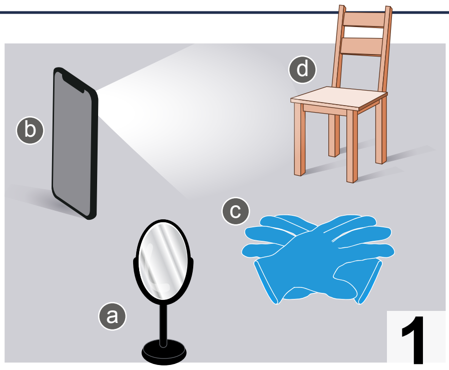


**What you may need for self examination:**

a) A  Mirror (on a stand)
b) A Torch or light source
c) A pair of gloves (optional)
d) A chair or stool for support

- Wash you hands throughly with soap and water to begin.

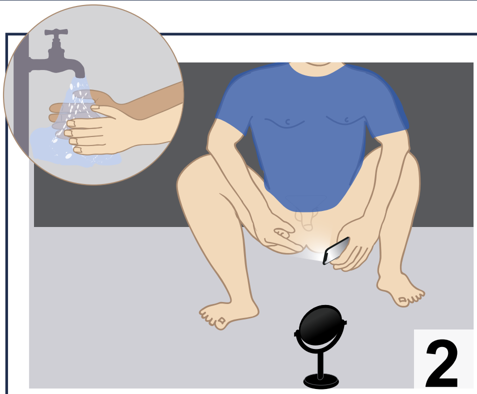


**Try to look at your anus:**

- You can use a mirror or smartphone app with selfie mode or mirror app to see your anus.
- Use a mirror with a stand or lean a mirror against an object and sit on the floor with your legs apart.
- Spread your butt cheeks to look at your anus.
- You can use a flash light for better lighting.

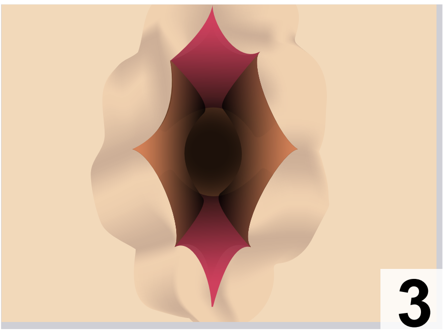


**Your anus upclose:**

- Think of your anal canal is a cylinder with four sections. You will use your finger to feel each section.

**Position yourself using one of the positions in the pictures shown (a - d) or a position that is comfortable for you to do anal exam**

- Choose the position that is most comfortable and gives you the best access to your anus.
- You can put on a glove if you prefer to use one.
- Put some lube or soap on your index finger or “pointing” finger.
- Feel the outside of the anal canal first.
- Then rest your finger for a second or two at the opening of your anus. It will help your anal relax.

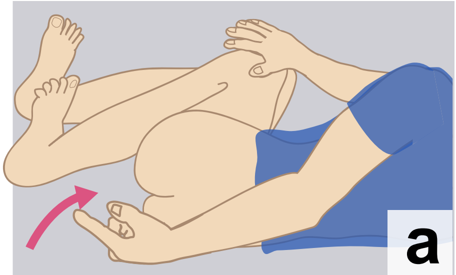


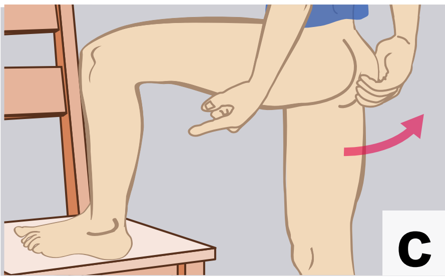


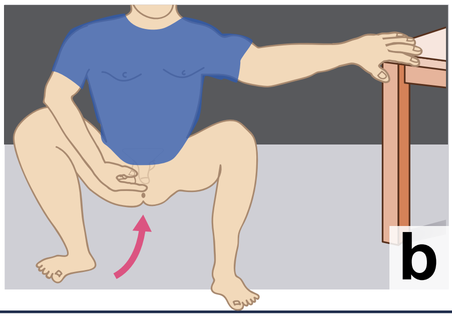


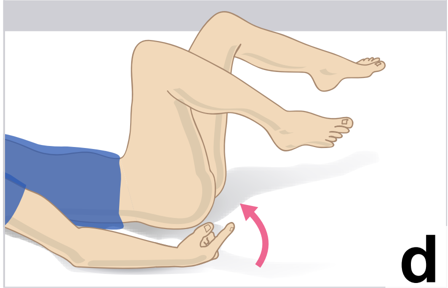


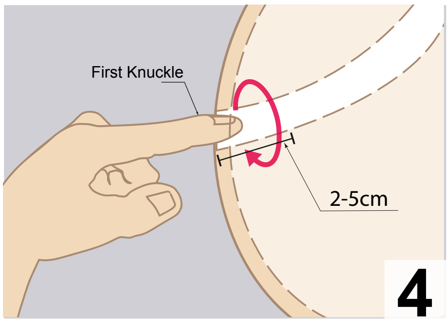


**Anal Canal:**

- Insert your index finger to your first knuckle. Feel around your anal canal in all four sections.
- Push your index finger to the second knuckle and feel as far as you can go around (360 degree)

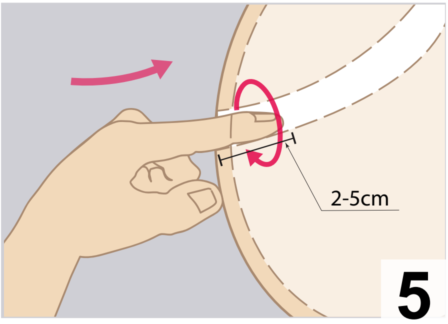


**Index Finger**

- Feel the lining of the wall. Feel for hard or soft lumps. Also note if there was bleeding, pus, pain associated with them.
- Pull your finger out when done.

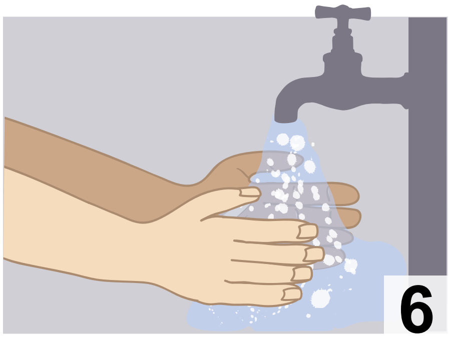


**Finish:**

- Dispose of the gloves if you’ve used them.
- Wash you hands throughly with soap and water.


**Document no:** SEASP001 **Version:** 2.0 **Issued by:** MSHC **Issue Date:** 29/07/2020
